# Thiophene-Based Covalent Triazine Frameworks as Visible-Light-Driven Heterogeneous Photocatalysts for the Oxidative Coupling of Amines

**DOI:** 10.3390/molecules29071637

**Published:** 2024-04-05

**Authors:** Manuel Melero, Urbano Díaz, Francesc X. Llabrés i Xamena

**Affiliations:** Instituto de Tecnología Química, Universitat Politècnica de València, Agencia Estatal Consejo Superior de Investigaciones Científicas, 46022 Valencia, Spain; mamegu@upvnet.upv.es

**Keywords:** covalent triazine frameworks, organic photocatalysis, benzylamine coupling

## Abstract

This study reports on a metal-free Covalent Triazine Framework (CTF) incorporating bithiophene structural units (TP-CTF) with a semicrystalline structure as an efficient heterogeneous photocatalyst under visible light irradiation. The physico-chemical properties and composition of this material was confirmed via different characterization solid-state techniques, such as XRD, TGA, CO_2_ adsorption and FT-IR, NMR and UV-Vis spectroscopies. The compound was synthesized through a solvothermal process and was explored as a heterogeneous photocatalyst for the oxidative coupling of amines to imines under visible light irradiation. TP-CTF demonstrated outstanding photocatalytic activity, with high conversion rates and selectivity. Importantly, the material exhibited exceptional stability and recyclability, making it a strong candidate for sustainable and efficient imine synthesis. The low bandgap of TP-CTF enabled the efficient absorption of visible light, which is a notable advantage for visible-light-driven photocatalysis.

## 1. Introduction

Photocatalysis [1] refers to light-driven catalytic reactions using solar power as a clean, sustainable and overall green energy source for resolving the serious energy crisis and environmental pollution. Several researchers have reported UV light usage to activate molecules in chemical transformations, although visible light is the ideal alternative green source to activate photocatalytic reactions. In recent years, Covalent Organic Polymers (COPs) incorporating structural photoactive building units [2] have been explored as novel heterogeneous photocatalysts to improve conventional methods in chemical industry. This is due to their good photostability, recyclability, light-harvesting properties and charge separation abilities through internal donor–acceptor pairs. Thus, COPs have shown excellent photocatalytic properties as promising platforms in the fields of chemistry and materials science, due to their electron donor–acceptor characteristics and π-conjugated systems arising from structural chromofores (e.g., triazines, pyrenes, benzothiazoles, porphyrines) typical of COPs.

Within the vast group of COPs, porous organic polymers [3,4,5,6,7] in particular have attracted much interest in recent decades due to their wide range of physicochemical properties, featuring high surface areas with accessible cavities for the encapsulation or insertion of chemical compounds. These materials have inspired the research community to improve and design new highly porous materials with tunable chemical and structural properties. Covalent Organic Frameworks (COFs) [8,9,10] are a new class of crystalline organic porous materials composed via the self-assembly of periodically arranged organic building units of light-weight elements, such as B, N, O, Si, P or S, connected by covalent bonds to form ordered 2D or 3D polymer networks containing polygonal uniform channels with different skeletons (imines, boronates, esters, ketoenamines, etc.) and specific pore sizes. Among the large family of COFs, Covalent Triazine Frameworks (CTFs) have recently been reported as good heterogeneous photocatalysts [11,12].

CTFs [13,14,15,16] are a subclass of COFs formed by structural triazine rings connected through covalent bonds into an extended porous framework with promising characteristics, including semi-crystallinity, high surface areas, physicochemical and thermal stability and a high nitrogen content. Thanks to these specific properties, CTFs have great potential in a wide range of applications, such as gas separation, energy and gas storage [17,18], as well as photo/electro/thermo-catalysis [19,20,21]. CTFs are a new type of nitrogen-rich porous material with a tunable molecular structure due to the variety of heteroatoms (N, P, S or F) that can be introduced in the linkages connecting the triazine rings. In this way, the introduction of structural heteroatoms provides modulated structures and multiple active sites that can be further used in post-modification methods to improve their characteristics. In particular, CTFs based on thiophene and bithiophene building blocks [22,23,24,25] are interesting materials with exciting properties. Thiophene derivatives can be found in several natural and synthetic compounds with numerous applications in biological and molecular science areas. Thiophene is a stable π-aromatic heterocyclic compound consisting of four carbon atoms and one sulfur atom in a five member-ring, with a well-known chemistry due to its aromaticity and easy and chemically controlled substitution in alpha or beta positions under mild reaction conditions. Thiophene derivatives [26] containing π-systems have rigid, electron-rich, flat delocalized structures and high charge transport properties, making them promising π-bridges and donors to construct conjugated and low-band-gap organic semiconductors for organic field-effect transistors (OFETs), organic light-emitting diodes (OLEDs) and organic photovoltaics (OPVs). In fact, the extended π-conjugation favors charge migration and separation capabilities, offering an improved route for the charge transport. Furthermore, thiophenochemistry remains of great research interest due to the easy improvements in derivatization substitution methods, as well as in the development of new sustainable synthesis methods.

Owing to the intrinsic properties mentioned above, several COFs [27,28,29] containing various photoactive structural moieties (triazines, pyrroles, pyridines, naphtalenes) have been reported as efficient heterogeneous photocatalysts [30,31] in a number of organic transformations [32,33], such as selective photo-oxidation and photo-reductions [34,35,36,37], selective coupling reactions [38], photo-mediated controlled radical polymerizations or the synthesis of linear and cyclic carbonates from CO_2_ and epoxides [39]. Furthermore, the incorporation of thiophene units into COF structures enhances their photocatalytic reactivity by broadening light absorption, improving charge separation and migration, facilitating redox reactions and enabling the precise tuning of electronic properties. These advantages make thiophene-based COFs promising materials for visible-light-driven heterogeneous photocatalysis, such as in the oxidative coupling reactions of amines.

Imines and their derivatives are valuable building block intermediates for the synthesis of fine chemicals, pharmaceuticals and biochemical inhibitors. They are often found in natural and biologically active products, which are usually prepared through the condensation of aldehydes and ketones or the hydroamination of alkynes with amines. However, most of the current available methods rely on the catalytic activity of rare metal catalysts, such as Co, Ru, Mn, etc. [40]. For this reason, new sustainable strategies for imine production, such as the oxidative coupling of amines [41,42], provide an attractive alternative to the traditional synthetic methods that require an acid catalyst and produce large amounts of chemical wastes. Moreover, the research of new metal-free heterogeneous catalysts for oxidative coupling reactions become an important way to obtain an efficient imine synthesis method.

**Scheme 1:**
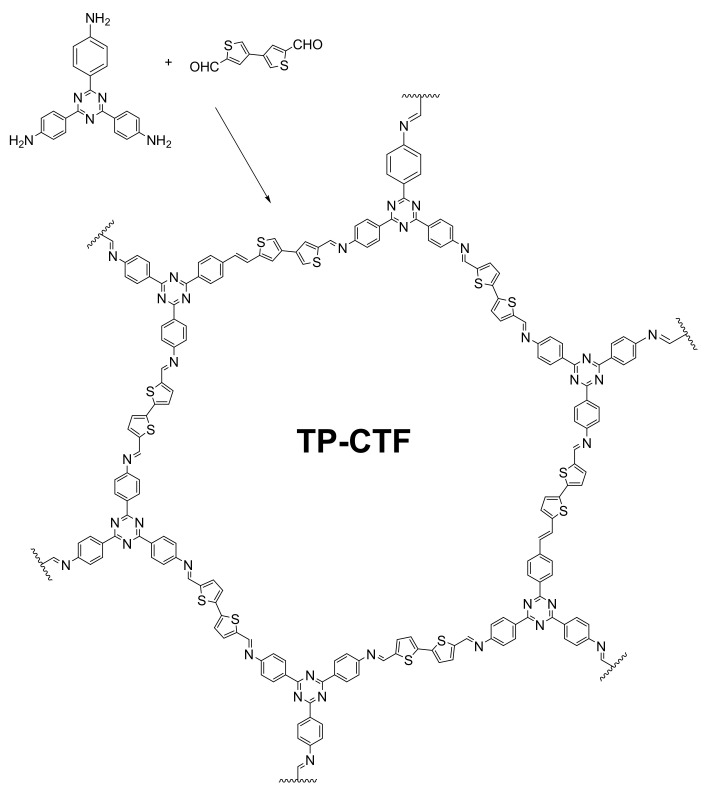
Schematic illustration of the synthetic procedure and the idealized structure of the obtained CTF.

In this work, we have prepared and characterized a metal-free CTF containing bithiophene structural moieties (hereafter TP-CTF) as a heterogenous photocatalyst for the oxidative coupling of various amines to imines, with a wide scope of substrates, showing stability, high conversions and good recyclability (Scheme 1). The TP-CTF-obtained material was profusely studied [43], with its physico-chemical properties and composition being confirmed using different characterization solid-state techniques, such as XRD, TGA, CO_2_ adsorption and FT-IR, NMR and UV-Vis spectroscopies.

## 2. Results and Discussion

### 2.1. Synthesis and Characterization

In this study, the TP-CTF photocatalyst was synthesized by reacting 4,4′,4″-(1,3,5-triazine-2,4,6-triyl)trianiline and 2,2′-bithiophene-5,5′-dicarboxaldehyde in mesitylene/dioxane (1:2) at 120 °C, following the procedure detailed in the Section 3 via solvothermal synthesis. Acetic acid was used as a Brønsted acid catalyst to accelerate the imine coupling reaction by condensing polyfunctional aldehydes and amines at an elevated temperature in a mixture of organic solvents (Scheme 1). The coupling reaction was complete within 72 h, and the resulting solid was isolated via filtration and washed with THF, MeOH and acetone in a Soxhlet extractor to afford an orange powder with a 90% yield for TP-CTF. Elemental analysis was used to determine the experimental C:S and C:N molar ratio of the TP-CTF. As shown in Table S1, the data show an excellent coincidence with the values expected for the stoichiometric compound, which confirms the quantitative assembly between starting building units.

**Figure 1:**
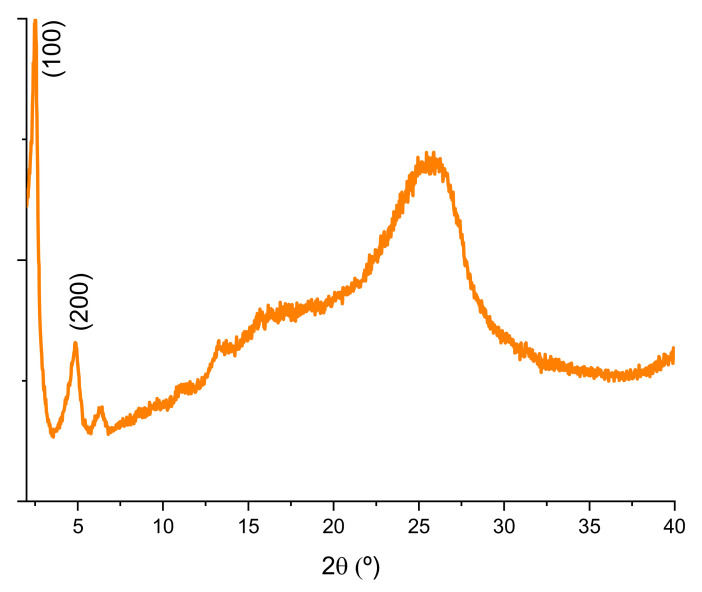
XRD diffractogram of TP-CTF material.

According to the X-ray powder diffractogram shown in Figure 1, TP-CTF was obtained as a semicrystalline CTF, with a peak distribution similar to that found in many lamellar COFs [44,45,46,47,48,49,50]. One of the key characteristics of 2D CTFs with bithiophene units is the presence of peaks in the XRD pattern that correspond to the interlayer spacing or *d*-spacing. The interlayer spacing is the distance between adjacent layers in the laminar structure of the CTF. These individual layers are formed through the covalent bonding of triazine rings and bithiophene units [51,52], creating a repeating two-dimensional framework. The XRD peaks related to interlayer spacing are a direct reflection of the stacking of these layers. The diffraction pattern often exhibits sharp, well-defined peaks at specific 2θ low-angles, evidencing the periodic nature of this characteristic interlayer stacking.

The specific 2θ angles at which these peaks appear are indicative of the distance between adjacent layers in the CTF structure, which is of paramount importance, as it affects various properties and applications, particularly in gas adsorption and separation processes. A prominent feature in CTF XRD patterns is the (100) facet of a primitive hexagonal lattice, which corresponds to the spacing between consecutive layers, featuring an eclipsed (AA) stacking mode with a *P*6/m space group, instead of staggered (AB) stacking mode [53]. The exact position of the (100) peak varies depending on the thickness of individual layers and the presence of solvent molecules in the interlayer space and typically falls in the range of 3.5° to 6° 2θ. The (200) diffraction peak, clearly observed in the X-ray patterns, represents the second-order reflection of the interlayer spacing, confirming that the lamellar spatial distribution and interlayer space is regularly maintained along the 2D COF-type structure. It usually appears at a higher 2θ angle than the (100) peak, often in the range of 6° to 12° 2θ.

**Figure 2:**
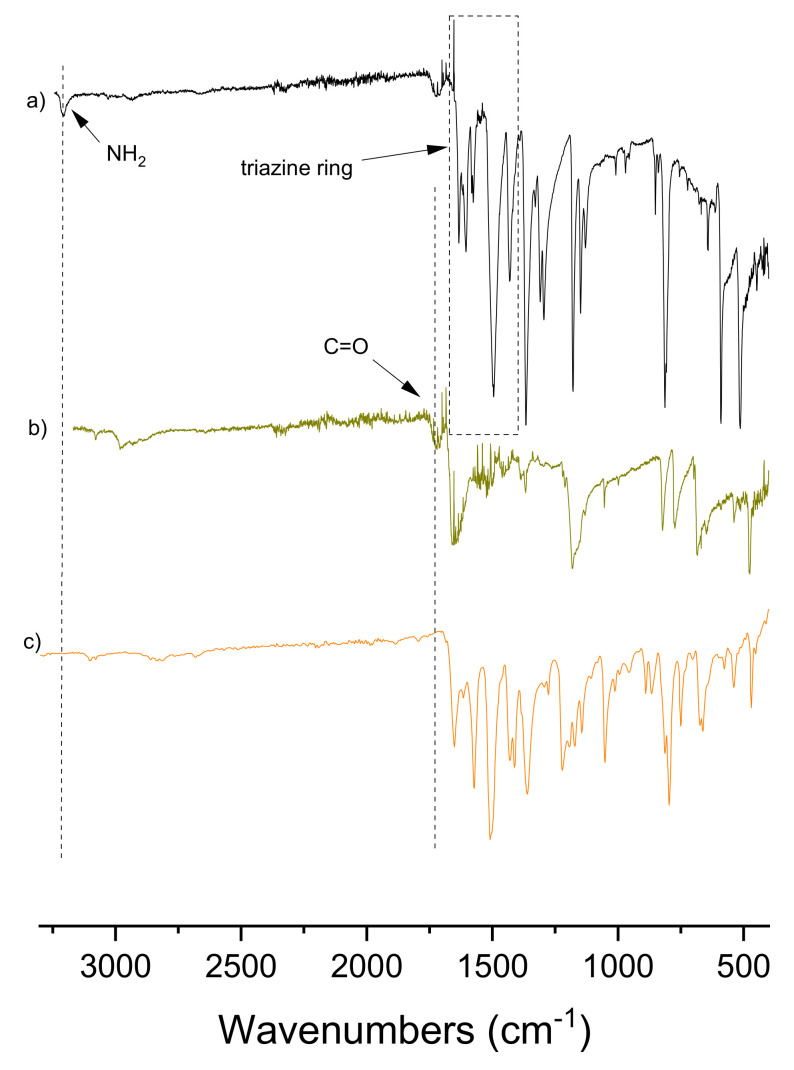
FT-IR spectra of starting materials 4,4′,4″-(1,3,5-triazine-2,4,6-triyl)trianiline (a), 2,2′-bithiophene-5,5′-dicarboxaldehyde (b) and the TP-CTF photocatalyst (c).

**Scheme 2:**
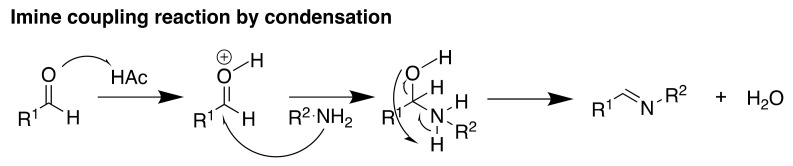
Imine coupling reaction via condensation between an aldehyde and a primary amine to form the TP-CTF material.

Figure 2 provides a comparative analysis of the FT-IR spectra of TP-CTF and its constituent monomers, shedding light on the structural transformations that occur during the polymerization process. A prominent feature observed in the FT-IR spectra is the transformation of functional groups, which is discernible through the decrease in intensity or even the complete disappearance of the band around 3200 cm^−1^, corresponding to the NH_2_ group of the triazine precursor, and the disappearance of the C=O absorption band of the aldehyde precursor, typically found at around 1650 cm^−1^. These changes evidence the condensation reaction between the amine and aldehyde groups of the precursor monomers into imine linkages, a crucial aspect of the polymerization process (Scheme 2). The emergence of characteristic imine bonds (-C=N) in TP-CTF is expected in the spectral region spanning from 1600 to 1640 cm^−1^. However, it is essential to exercise caution when assigning the imine band, as the triazine bands of CTFs are in close proximity to this spectral region and can potentially overlap. This spectral overlap adds a layer of complexity to the assignment process, underscoring the need for meticulous analysis to accurately identify and attribute the imine-related bands within the FT-IR spectra.

**Figure 3:**
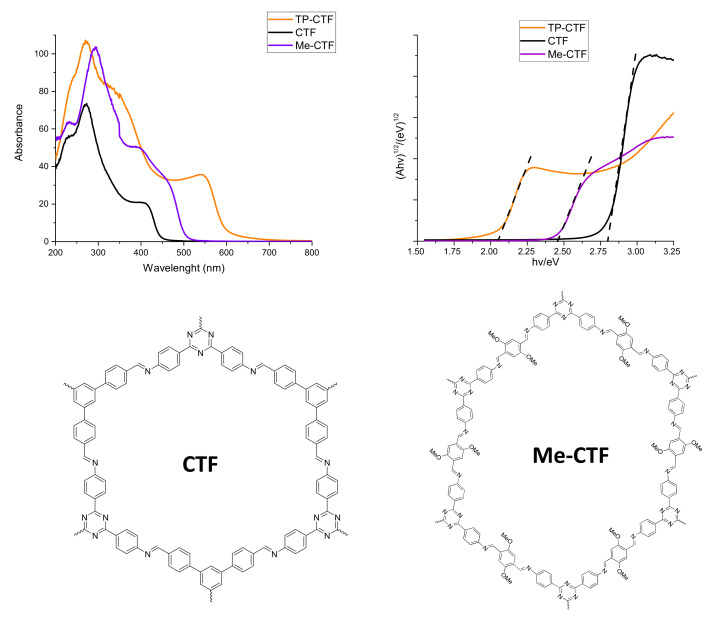
UV spectra of photocatalysts (**top-left**) and corresponding Tauc plots (**top-right**). The structures of the two compounds (CTF and Me-CTF) used for the comparison are also shown (**bottom**).

Light absorption properties of the TP-CTF photocatalyst were investigated by means of UV-Vis spectroscopy, and the corresponding spectrum is presented in Figure 3. The TP-CTF photocatalyst featured strong absorption bands at about 280, 380 and 550 nm, indicating that the solid can efficiently absorb solar energy in the UV and visible regions. According to the corresponding Tauc plot [53] shown in Figure 3, the optical bandgap (E_g_) of TP-CTF is 2.1 eV. Clearly, the absorption in the visible region of TP-CTF is not related to the triazine but to the bithiophene moieties in the structure. Other CTF-type materials lacking these thiophene units (such as CTF and Me-CTF shown in Figure 3 for comparison) presented much higher bandgaps (2.8 and 2.5 eV, respectively) and do not significantly absorb in the visible region. This is an important feature with respect to the potential activity of TP-CTF as a visible light drive photocatalyst.

**Figure 4:**
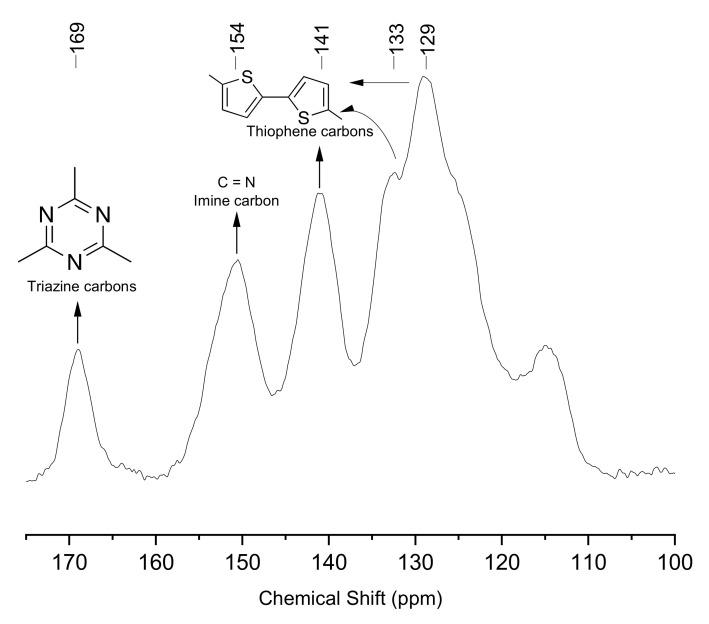
^13^CP/MAS NMR spectrum and peak assignment of TP-CTF material.

CTFs in general are not soluble in common organic solvents due to their high chemical stability arising from the assembly by covalent bonds. Therefore, solid-state CP-MAS ^13^C NMR spectroscopy was used to characterize the photocatalyst. The corresponding spectrum of TP-CTF shown in Figure 4 presents chemical shifts at 123, 125, 139 and 150 ppm collectively assigned to all sp^2^ carbon atoms associated with structural aromatic moieties. In addition, chemical shifts corresponding to N=C-N triazine groups (169 ppm) and thiophene carbons (129, 133 and 144 ppm) were also observed in the NMR spectrum. Also, of note, a chemical shift corresponding to the C=N imine linkages between triazine and bitiophene units was observed at 154 ppm. Finally, an additional peak was observed at 115 ppm in the spectrum, where the chemical shift corresponding to the C-NH_2_ groups of the triazine is expected. This indicates that a small amount of free -NH_2_ groups still remains in TP-CTF, which is associated with structural defects.

The thermal stability of CTFs was evaluated via thermogravimetric analysis (TGA) under air flux, as shown in Figure S3. The degradation of organic building units started in the 450 to 650 °C range, evidencing the high thermal stability of TP-CTF thanks to the intrinsic stability of aromatic coordinated units present in the organic framework. The textural properties of TP-CTF were analyzed via CO_2_ adsorption at 0 °C up to 1 bar, as shown in Figure S5. The corresponding surface areas were estimated from the isotherms by using the Dubinin–Astakov equation [54]. The measured CO_2_ capacities (at 1 bar) corresponded to a calculated surface area of 235 m^2^ g^−1^ for TP-CTF. Thus, the porosity of this material is relatively low, in comparison with traditional imine-based COFs, like TpPa1-COF [55], which are typically in the range 500–1600 m^2^ g^−1^ depending on the activation temperature. It is interesting to note that TP-CTF does not adsorb any sensible amount of N_2_ at a low temperature (77 K). Therefore, CTFs containing bithiophene units could have a high potential for the selective separation of gas mixtures, e.g., N_2_/CO_2_, which is currently being explored by our research group.

**Figure 5:**
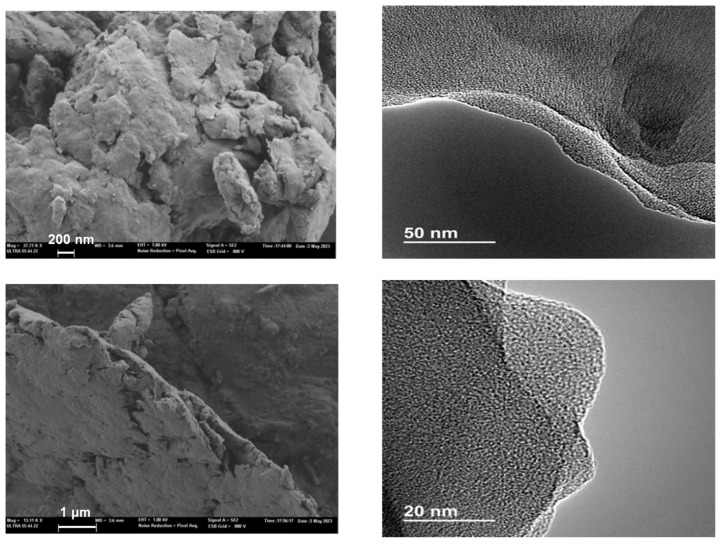
SEM (**left**) and TEM (**right**) images of TP-CTF material. Scale bars are included in the micrographs.

The morphology and microstructure of the as-prepared photocatalyst was characterized via scanning (SEM) and transmission (TEM) electron microscopy, and the images of TP-CTF are shown in Figure 5. The material showed a layered stacking morphology with a micron level size. In order to reveal the eventual ordering of TP-CTF, TEM images were obtained. A close examination of TP-CTF evidenced the layered stacking morphology of this compound. The individual layers of the CTF were observed to be superimposed on top of each other. This laminar arrangement is a direct result of the covalent bonds connecting the building units within each layer, corroborating the results obtained from XRD patterns. It creates a unique layered structure, similar to the pages of a book stacked on one another. This layered morphology plays a vital role in the material’s properties, particularly its porosity, surface area and accessibility. Additionally, TEM images revealed a degree of disorder within the TP-CTF structure, especially when different layers or sections overlap. This disorder is a consequence of the intricate process of layer-by-layer deposition during CTF formation. As successive layers are deposited on top of each other, they may not align perfectly, leading to variations in the positioning of atoms and functional groups. This structural disorder can have important implications for the material’s properties, including gas adsorption, catalytic activity and more.

### 2.2. Photocatalytic Coupling of Amines

**Scheme 3:**
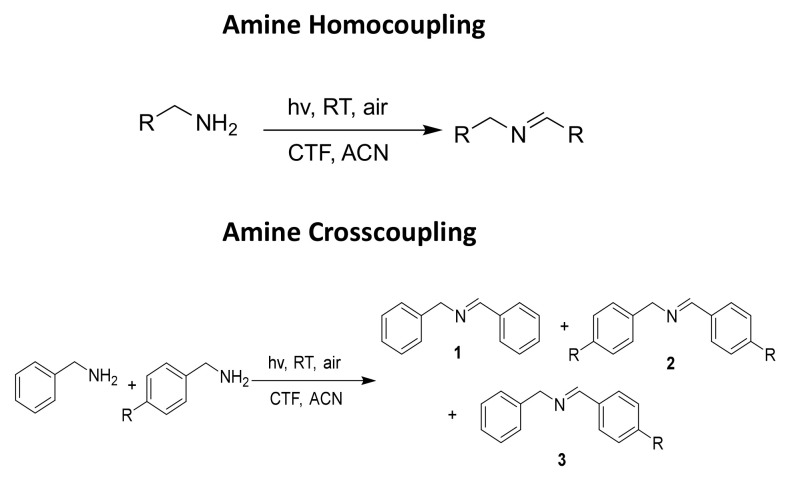
Oxidative aerobic amine homo- and cross-coupling photoreaction.

**Figure 6:**
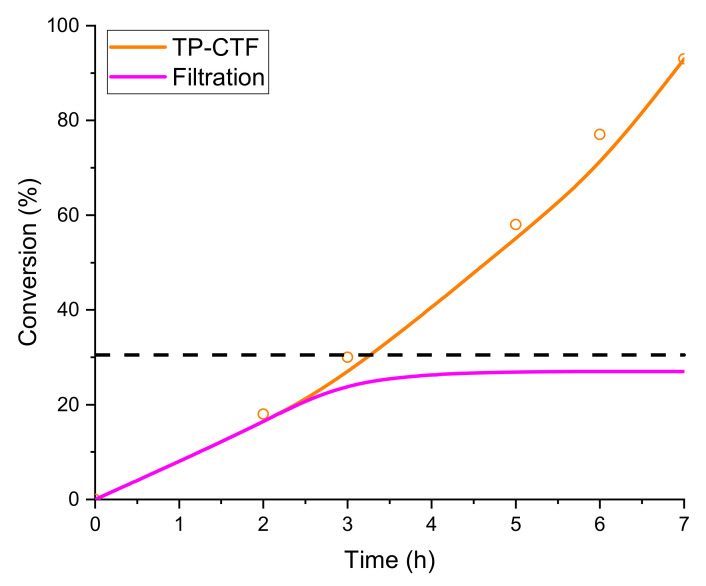
Time-conversion of benzylamine to *N*-benzylidenebenzylamine using TP-CTF as the photocatalyst. The dashed line indicates the conversion at which the catalyst was withdrawn from the reaction in the hot filtration experiment (see below).

In order to evaluate the potential photocatalytic activity of thiophene-based CTF as a heterogeneous photocatalyst, we have considered the aerobic (homo- and cross-) coupling reaction of amines [56] at room temperature and under visible light irradiation (λ_irr_ > 400 nm) over TP-CTF (Scheme 3). First, we evaluated the homocoupling of benzylamine as a test reaction. The results showed that 100% conversion of imine was achieved after 7 h irradiation at room temperature when TP-CTF was used as the photocatalyst (entry 2 in Table 1). The product of the homocoupling reaction of benzylamine, *N*-benzylidenebenzylamine, was detected as the sole product, as confirmed via GC and GC-MS. The time-conversion plot obtained for the oxidative coupling reaction of benzylamine using TP-CTF as photocatalyst is shown in Figure 6.

We have recently studied in detail [57] the mechanism of the visible-light-driven photocatalytic coupling of benzylamine over MIL-125-NH_2_ by combining catalytic tests, IR spectroscopy and density functional calculations. According to this mechanism, the reaction proceeds through the oxidation of benzylamine to benzaldehyde, followed by a nucleophilic attack by a second benzylamine molecule and dehydration to yield the corresponding imine. The role of the photocatalyst consists of the transfer of a photogenerated electron to O_2_, forming a O_2_^•−^ radical anion. All the data obtained in the present work suggest that the same mechanism also applies to the TP-CTF photocatalyst.

Other covalent triazine frameworks lacking the bithiophene moieties, namely CTF and Me-CTF [36,57,58], were used for a comparison, and the data obtained are also summarized in Table 1. Although these thiophene-free compounds also exhibited some potential in the considered photocatalytic reaction, they do not perform as well as the TP-CTF material due to their higher bandgap, which significantly decreases the light absorption in the visible region (compare entry 2 with entries 4 and 5 in Table 1). The catalytic performance of CTF and Me-CTF is particularly noteworthy as it predominantly arises from the inherent properties of the triazine rings within their structures. Both CTF and Me-CTF primarily rely on these triazine units to facilitate the amine-to-imine oxidative coupling reactions under visible light irradiation. CTF and Me-CTF efficiently promote the conversion of amines to imines with a 52% conversion rate. This suggests that the triazine moieties, with their intrinsic catalytic activity, serve as the driving force for this reaction, highlighting the pivotal role of the triazine units in CTF’s catalytic performance [59,60,61,62,63,64,65]. The presence of thiophene units may further enhance the catalytic activity, as demonstrated by TP-CTF, but it is not the sole factor.

Meanwhile, no conversion was observed when the experiment was carried out in the absence of any catalyst or under dark conditions (entries 1 and 3). This outcome highlights the fundamental role played by the photocatalyst in driving the chemical transformation. In the absence of light, the activation process is hindered, emphasizing the essential nature of the visible light source in initiating the catalytic cycle. Furthermore, the minimal conversion in the blank reaction underscores the inefficacy of the reaction in the absence of the catalyst, reinforcing the catalytic material’s indispensable role in facilitating the aerobic coupling of amines to imines under visible light irradiation. This discussion reaffirms the photoreactivity of the CTF catalyst and its pivotal function in promoting the selective and efficient transformation of amines to imines in an environmentally friendly manner [66].

Encouraged by the good results obtained with TP-CTF, the scope of the reaction was further investigated for the aerobic homo- and cross-coupling of other benzylamine substrates using TP-CTF as the photocatalyst under visible light irradiation.

Quantitative and fully selective imine yields were attained within 7 h of irradiation for the homocoupling of 4-fluorobenzylamine and phenetylbenzylamine (Table 1 entries 6 and 7). The cross-coupling reaction between benzylamine and 4-fluorobenzylamine also achieved full conversion, with the considerable selectivity of 59% for the cross-coupling product. This probably indicates that the reaction rate of the two homo-coupling reactions and that of the cross-coupling are similar, so it is unavoidable that all three reactions take place simultaneously. Nevertheless, the aerobic photocoupling of amines provides an interesting route for obtaining non-symmetrical imines with reasonably good yields.

**Figure 7:**
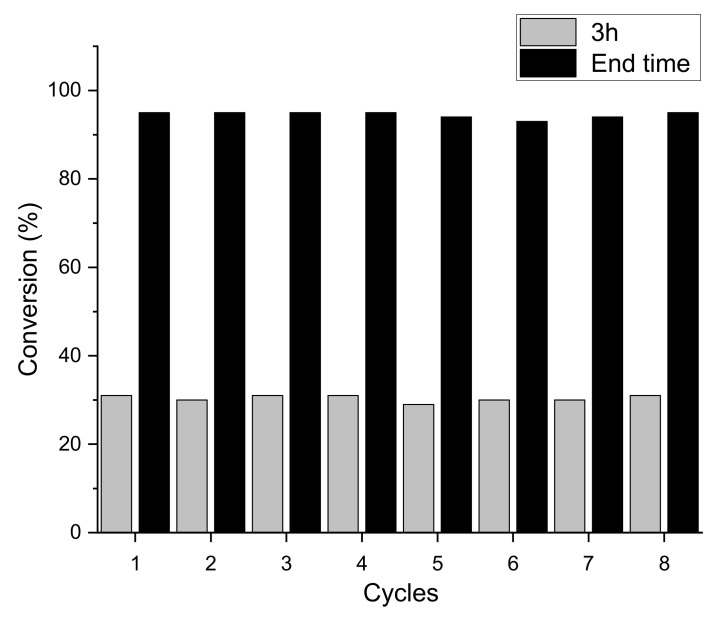
Recyclability of TP-CTF catalyst for the benzylamine coupling reaction at 3 h and the end time (7 h).

One of the remarkable features of TP-CTF is its outstanding stability and recyclability, which significantly contribute to its sustainable and eco-friendly catalytic potential. The stability and recyclability of TP-CTF photocatalyst were examined in the aerobic oxidation of benzylamine homocoupling. After each catalytic run, TP-CTF was filtered and washed with acetonitrile and then dried at 100 °C before the next run. In a series of consecutive catalytic cycles, TP-CTF consistently maintained a high level of conversion of 95% and full selectivity to the homocoupling product for at least 8 consecutive catalytic cycles, as shown in Figure 7, with minimal performance degradation. These observations underscore the robustness and longevity of TP-CTF as a heterogeneous photocatalyst, rendering it suitable for long-term and repetitive use in the aerobic oxidative coupling of amines.

The remarkable catalytic stability of TP-CTF constitutes a highly advantageous feature for industrial applications, where the longevity and consistent performance of a catalyst play pivotal roles. In order to provide a comprehensive assessment of its stability and catalytic activity, a detailed X-ray diffraction and elemental analysis was conducted following its first use in the catalytic reaction. In the X-ray diffraction patterns (see Figure S2), a subtle decrease in the intensity of characteristic peaks was observed. However, this decrease did not compromise the overall semicrystalline structural chemistry of TP-CTF, indicating that its fundamental structural integrity remained intact.

In tandem with X-ray diffraction, elemental analysis was conducted, focusing on carbon and sulfur (C:S) and carbon and nitrogen molar ratios (C:N), as summarized in Table S1. The results confirmed that TP-CTF maintained its initial C:S and C:N ratios, underlining its robust chemical composition. These findings provide valuable insights into the structural and chemical resilience of TP-CTF. Moreover, the use of TP-CTF aligns with the principles of green chemistry, emphasizing the importance of catalyst recyclability and longevity in reducing waste and promoting a more sustainable chemical industry.

During the course of the hot filtration experiment, which involved filtering the reaction slurry at the reaction temperature, a significant observation emerged regarding the catalytic behavior. As the reaction continued and the catalyst remained in contact with the reaction mixture, a notable increase in conversion was observed. However, the turning point occurred when the hot filtration process was initiated, which marked a pivotal moment in the reaction’s progress. What became apparent was that once the catalyst was separated from the reaction mixture via filtration at an intermediate stage of the reaction (as depicted in Figure 6), the conversion abruptly ceased to increase any further. This observation hinted at a strong correlation between the catalyst and the reaction progress, signifying the critical role played by the catalyst in facilitating the reaction.

The subsequent analysis of the filtrate from the hot filtration experiment revealed an absence of sulfur, as confirmed through elemental analysis. This outcome is particularly significant, as it indicates that no active sites from the catalyst were leached into the reaction solution during the course of the reaction. The absence of sulfur detection underscores the robust integrity of the catalyst and its ability to maintain its structural and chemical integrity throughout the catalytic process. Moreover, this finding provides substantial evidence supporting the heterogeneous nature of the catalytic system. The lack of leaching and the stable catalyst structure further reinforce the argument that TP-CTF is an efficient and durable photocatalyst, capable of driving the reaction without being consumed or compromised in the process.

## 3. Experimental Section

### 3.1. Reagents and Chemicals

4,4′,4″-(1,3,5-triazine-2,4,6-triyl)trianiline and 2,2′-bithiophene-5,5′-dicarboxaldehyde were purchased from TCI Europe and ABCR, respectively. Benzylamine, 4-fluorobenzyylamine, phenetylamine, 1,4-dioxane and mesitylene were purchased from Sigma Aldrich. Acetonitrile and PTFE syringe filters were purchased from Scharlab.

### 3.2. Synthesis of TP-CTF

A Schlenk flask tube with vacuum valve was charged with 4,4′,4″-(1,3,5-triazine-2,4,6-triyl)trianiline (106 mg, 0.3 mmol), 2,2′-bithiophene-5,5′-dicarbaldehyde (99 mg, 0.45 mmol), 1.0 mL of mesitylene, 2.0 mL of dioxane and 0.5 mL of 6 M aqueous acetic acid. The whole content was sonicated for 10–12 min at room temperature in order to obtain a homogenous dispersion. The tube was then flash frozen at 77 K (liquid N_2_ bath) and degassed through three freeze-pump-thaw cycles, after which the flask was charged with N_2_ through the vacuum valve. The tube was sealed off under a vacuum and then heated at 120 °C for 3 days without stirring. A red colored precipitate was collected via centrifugation and washed with anhydrous methanol and finally with anhydrous acetone. The powder collected was then washed in a Soxhlet extractor with THF (12 h) at 90 °C and then dried at 100 °C for 2 h to give a dark-red-colored pure TP-CTF powder.

### 3.3. Catalyst Characterization

The C, H and N content of the sample TP-CTF was determined with a Carlo Erba 1106 elemental analyzer. Thermogravimetric and differential thermal analysis (TGA-DTA) were performed in an air stream with a Mettler Toledo TGA/SDTA 851E analyzer. Solid-state MAS-NMR spectra were obtained at room temperature under magic angle spinning (MAS) in a Brucker AV-400 spectrometer. IR spectra were determined with a Bruker Tensor 27 FT-IR spectrometer. Adsorption isotherms were measured in a Micromeritics ASAP 2010 instrument using approximately 200 mg of the adsorbent placed in a sample holder that was immersed in a liquid circulation thermo-static bath for precise temperature control. Before each measurement, the sample was treated overnight at 673 K under a vacuum. CO_2_ adsorption isotherms were then acquired at 273 K. UV–Vis Diffuse Reflectance Spectroscopy (DRS) was performed using a Cary 5 spectrometer equipped with a Diffuse Reflectance accessory in the range between 190 and 800 nm. This technique was used to determine the band gap value of the samples, Eg, related to the absorption coefficient using the Tauc equation. Catalytic results were obtained in a GC with a HP-5 column.

### 3.4. Photocatalytic Experiments

#### 3.4.1. General Procedure for the Homo-Coupling of Benzylamine

In a typical run for photocatalytic activity test of TP-CTF, benzylamine (1.0 mmol), acetonitrile (2 mL) and TP-CTF (15 mg) were added to a 50 mL quartz cell. The reaction mixture was stirred at room temperature and ambient air pressure and irradiated with visible light (1000 W Xe lamp with a UV filter, λ_exc_ > 420 nm). The reaction products were analyzed via GC (Shimadzu 2010, equipped with a FID detector and a HP5 30 m × 0.25 mm × 0.25 μm column) based on sample aliquots taken at fixed time intervals and using dodecane as an internal standard. After each catalytic cycle, the solid was filtered and washed with acetonitrile (3 × 10 mL), then dried at 100 °C in a vacuum pump and used directly in a consecutive cycle.

#### 3.4.2. General Procedure for the Cross-Coupling of Amines

Analogous to the homocoupling experiment, benzylamine (1.0 mmol) and 4-fluorobenzylamine (1.8 mmol), acetonitrile (5.6 mL) and TP-CTF (15 mg) were added to a 50 mL quartz cell. The reaction mixture was irradiated at room temperature under the same conditions as above, and the products were analyzed via GC using the same setup. After each cycle, the catalyst was filtered and washed with acetonitrile (3 × 10 mL), then dried at 100 °C in a vacuum pump and used directly in a consecutive cycle.

## 4. Conclusions

In summary, a bithiophene-based CTF compound named TP-CTF has been prepared via solvothermal synthesis and evaluated as a potential photocatalyst for the selective transformation of amines to imines at room temperature under visible light irradiation. Characterization data confirm the effective assembly of the starting building units used in the synthesis. An XRD diffractogram showed a semicrystalline structure, similar to that found in many lamellar COFs. The TP-CTF photocatalyst exhibited excellent activity, selectivity and recyclability for the homo- and cross-coupling reaction of amines, where quantitative yields of the target imine were obtained through an aerobic oxidative mechanism in an air atmosphere.

The incorporation of bithiophene units into the TP-CTF structure offers several advantages that contribute to its superior performance as a photocatalyst for the oxidative coupling of amines. Bithiophene moieties possess favorable electronic properties, such as π-conjugation and delocalization of electron density, which can facilitate charge separation and transfer processes during photocatalytic reactions. Additionally, the presence of sulfur atoms in the bithiophene units may enhance the adsorption and activation of reactant molecules, thus promoting efficient catalytic turnover. Furthermore, the extended conjugated system provided by the bithiophene skeleton together with the presence of triazine builder units can lead to improved light absorption properties, thereby enhancing the utilization of solar energy for photocatalysis. This enhanced light-harvesting ability may contribute to the observed higher catalytic activity of TP-CTF compared to other CTFs lacking thiophene units.

Definitively, these new heterogeneous bithiophene-based photocatalysts can offer breakthroughs in the field of photocatalysis as promoted materials with high potential in a multitude of industrial reactions. This study not only presents TP-CTF as a promising photocatalyst but also underscores the potential of thiophene-based CTFs in catalytic applications, particularly in industrial processes, opening new avenues for sustainable and green chemistry. The findings pave the way for further research on utilizing such materials to address complex challenges in the chemical industry and environmental remediation.

## Figures and Tables

**Table 1:** Homo- and cross-coupling of different amines with TP-CTF as the photocatalyst *^a^*.

Entry	Catalyst	Substrate	Product	Conv (%)	Select (%)
1	Blank	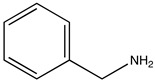	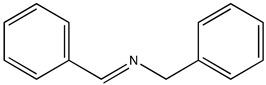	0	0
2	TP-CTF			100	100
3	TP-CTF *^b^*			0	0
4	CTF			52	100
5	Me-CTF			30	100
6	TP-CTF	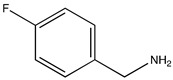	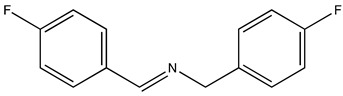	100	100
7	TP-CTF	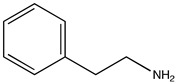	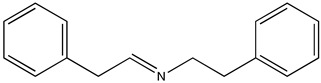	100	100
8	TP-CTF	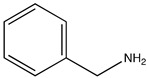 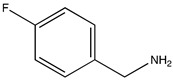	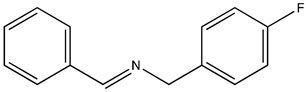	100	59

*^a^* Reaction conditions: benzylamine (1.0 mmol), acetonitrile (2 mL) and solid photocatalyst (15 mg), air atmosphere, room temperature, visible light irradiation (λ_irr_ > 700 nm), 7 h of reaction. *^b^* Same as in entry 2 but without irradiation.

## Data Availability

The original contributions presented in the study are included in the article/Supplementary Materials; further inquiries can be directed to the corresponding authors.

## Supplementary Materials

The following supporting information can be downloaded at: https://www.mdpi.com/article/10.3390/molecules29071637/s1, Synthesis of CTF-type materials; Table S1: Elemental analysis of TP-CTF; Figures S1 and S2: XRD patterns; Figures S3 and S4: TG analysis; Figure S5: CO_2_ adsorption isotherm.
