# Supplementary material for: Thiophene-Based Covalent Triazine Frameworks as Visible-Light-Driven Heterogeneous Photocatalysts for the Oxidative Coupling of Amines
Source: Molecules. 2024 Apr 5;29(7):1637. doi: 10.3390/molecules29071637 (PMC11013671; doi:10.3390/molecules29071637)
Supplement: Supplementary file 1 [file molecules-29-01637-s001.zip › molecules-2915929-supplementary.pdf]

Supporting Information

Thiophene based-Covalent Triazine Frameworks as Visible-light Driven Heterogeneous Photocatalysts for the Oxidative Coupling of Amines

M. Melero, U. Díaz*, F.X. Llabrés i Xamena*

Instituto de Tecnología Química, Universitat Politècnica de València, Agencia Estatal Consejo Superior de Investigaciones Científicas, 46022 Valencia, Spain.
E-mail: udiaz@itq.upv.es; fllabres@itq.upv.es

Contents

Characterization data

Synthesis of CTF-type materials ………………………………………………………………………………3-4

Table S1. Elemental analysis of catalyst ………………………………………………………………………4

Figure S1 and S2, XRD patterns of CTF-type materials ………………………………………………5

Figure S3 and S4. TG analysis experiments of CTF-type materials ………………………………6

Figure S5. CO_2 adsorption isotherm of TP-CTF material ……………………………………………6

Synthesis of CTF-type materials

CTF's synthesis

To a Schlenk flask tube with vacuum valve, 1,3,5-tris(4-formylphenyl)benzene (0.57 mmol, 1 eq) and 4´,4´´-(1,3,5-triazine-2,4,6-triyl)trianiline (0.57 mmol, 1 eq) were added, followed by o-dichlorobenzene:ethanol:DMSO (2:1.8:0.2, 10.0 mL) along the walls of the flask (to push down any remaining solids atop of the flask), and 6 M acetic acid (1 mL). The flask was sealed and sonicated at room temperature for 10 min. The reaction mixture was then degassed through three freeze-pump-thaw cycles, after which the flask was charged with N_2 through the vacuum valve. The flask was then allowed to stand in a 120°C pre-heated oil bath for three days without stirring. The flask was removed from the oil bath, cooled to room temperature and filtered through a Buchner funnel equipped with a filter paper. The solid was washed with THF (50 mL x 3) and acetone (50 mL x 3) to ensure all of the material is filtered from the flask to the Buchner funnel. The solid was washed in a Soxhlet extractor with THF for 24 h and then was collected and dried at 120°C for 24 h under vacuum.

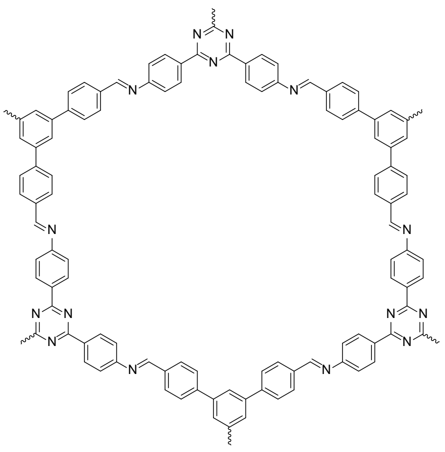

Me-CTF's synthesis

To a Schlenk flask tube with vacuum valve, 2,5-dimethoxybenzene-1,4-dicarboxaldehyde (0.86 mmol, 1.5 eq) and 4´,4´´-(1,3,5-triazine-2,4,6-triyl)trianiline (0.57 mmol, 1 eq) were added, followed by o-dichlorobenzene:n-buthanol (1:1, 10.0 mL) along the walls of the flask (to push down any remaining solids atop of the flask), and 6 M acetic acid (1 mL). The flask was sealed and sonicated at room temperature for 10 min. The reaction mixture was then degassed through three freeze-pump-thaw cycles, after which the flask was charged with N_2 through the vacuum valve. The flask was then allowed to stand in a 120°C pre-heated oil bath for three days without stirring. The flask was removed from the oil bath, cooled to room temperature and filtered through a Buchner funnel equipped with a filter paper. The solid was washed with THF (50 mL x 3) and acetone (50 mL x 3) to ensure all of the material is filtered from the flask to the Buchner funnel. The solid was washed in a Soxhlet extractor with THF for 24 h and then was collected and dried at 120°C for 24 h under vacuum.

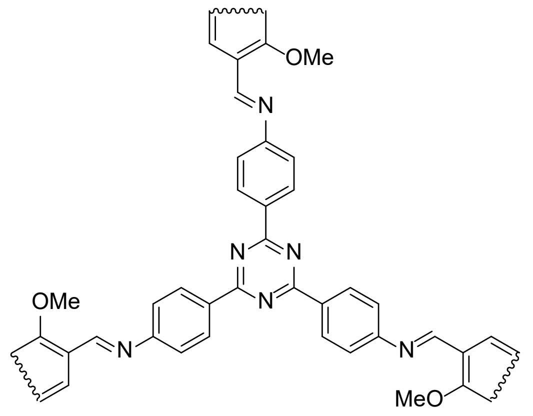

Elemental analysis of TP-CTF-type materials

Table S1. Elemental analysis of TP-CTF

Catalyst	N(%)	C(%)	H(%)	S(%)	C:S exp.	C:S calc.	C:N exp.	C:N calc.
Fresh TP-CTF	12.5	65.8	3.4	14.6	12.0	12.0	6.1	6.0
Recovered TP-CTF	13.0	69.1	4.3	15.0	12.0	12.3	6.1	6.2

XRD patterns of CTF-type materials

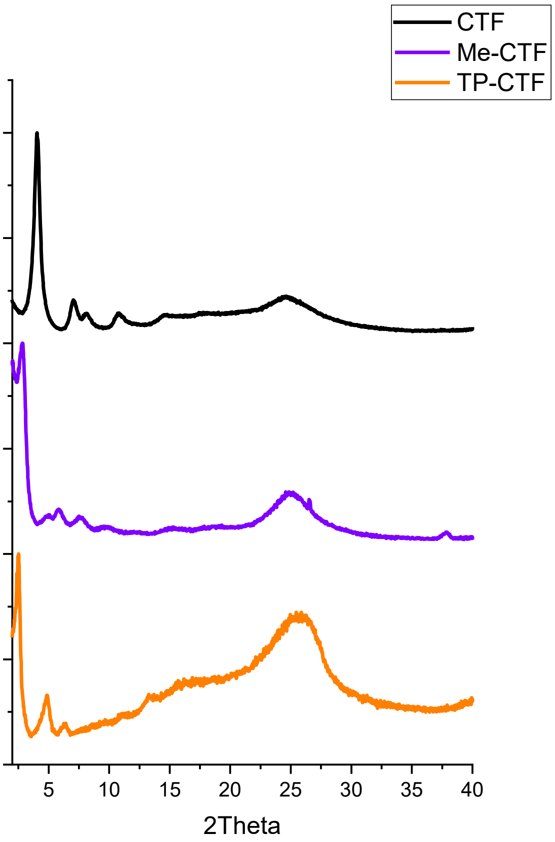

Figure S1. XRD patterns of CTF (a), Me-CTF (b) and TP-CTF (c) materials.

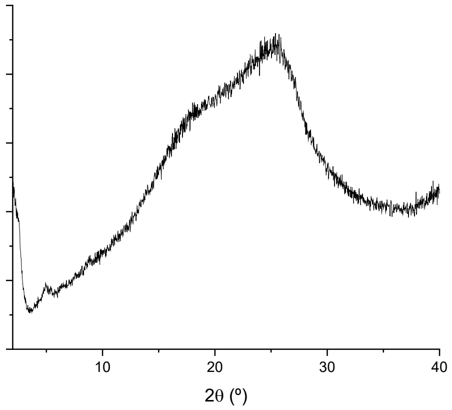

Figure S2. XRD pattern of recovered TP-CTF after 1st catalytic cycle.

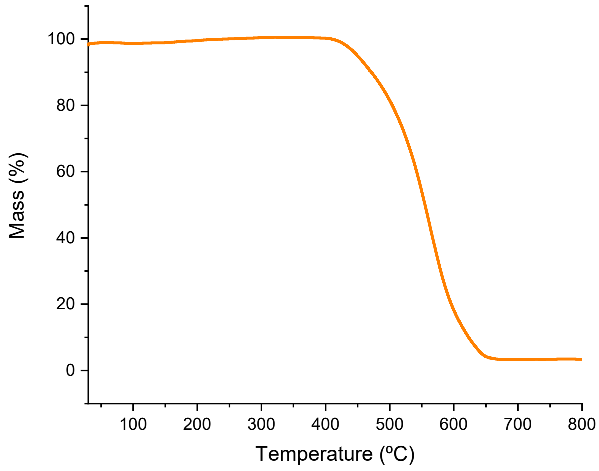

Figure S3. TG analysis of TP-CTF material.

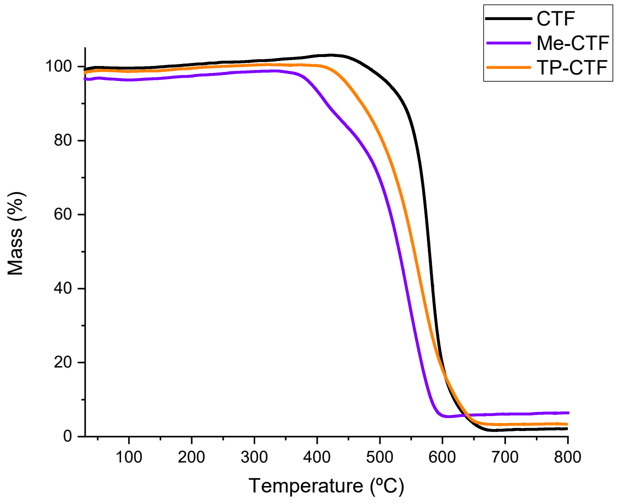

Figure S4. Comparative TG analysis of CTF (a), Me-CTF (b) and TP-CTF (c) materials.

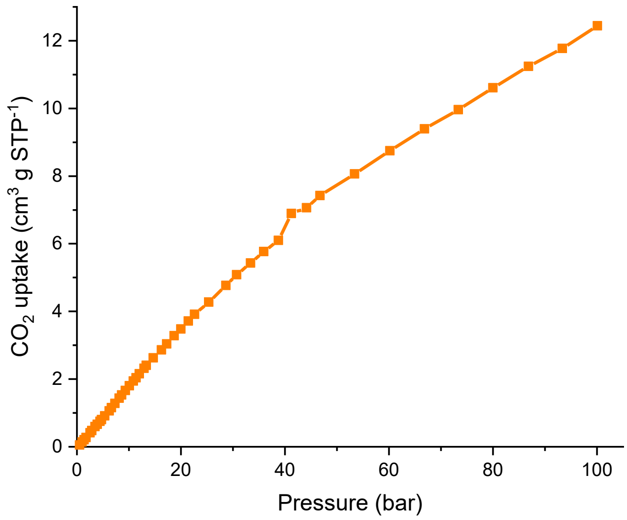

Figure S5. CO_2 adsorption isotherm of TP-CTF material.
